# Stereotype threat, gender and mathematics attainment: A conceptual replication of Stricker & Ward

**DOI:** 10.1371/journal.pone.0267699

**Published:** 2022-05-27

**Authors:** Matthew Inglis, Steven O’Hagan

**Affiliations:** 1 Centre for Mathematical Cognition, Loughborough University, Loughborough, United Kingdom; 2 School of Mathematics, University of Edinburgh, Edinburgh, United Kingdom; Tilburg University, NETHERLANDS

## Abstract

Stereotype threat has been proposed as one cause of gender differences in post-compulsory mathematics participation. Danaher and Crandall argued, based on a study conducted by Stricker and Ward, that enquiring about a student’s gender after they had finished a test, rather than before, would reduce stereotype threat and therefore increase the attainment of women students. Making such a change, they argued, could lead to nearly 5000 more women receiving AP Calculus AB credit per year. We conducted a preregistered conceptual replication of Stricker and Ward’s study in the context of the UK Mathematics Trust’s Junior Mathematical Challenge, finding no evidence of this stereotype threat effect. We conclude that the ‘silver bullet’ intervention of relocating demographic questions on test answer sheets is unlikely to provide an effective solution to systemic gender inequalities in mathematics education.

## Introduction

Mathematics education researchers have long been concerned that mathematics is experienced differently by men and women [1]. This concern is, in part, fueled by gender differences in post-compulsory participation rates in mathematical study and STEM careers [2].

One mechanism which some believe contributes to these observed gender differences in participation is *stereotype threat*. This account suggests that members of negatively stereotyped groups underperform when that stereotype is salient, perhaps because stereotype-related thoughts place an extra burden on stereotyped individuals’ cognitive resources [3]. For example, Steele and Aronson [4] found that black participants underperformed on laboratory tests of verbal ability compared to white participants, but only when reminded of negative stereotypes concerning race and intelligence. Similarly, Spencer, Steele and Quinn [5] found that women performed worse on a laboratory mathematics test than men, but only when they were told that the test usually revealed gender differences in achievement. Subsequently many similar lab-based studies have been conducted: a meta-analysis of 47 such studies showed that women, on average, underperform on laboratory mathematics tests by 0.22 standard deviations when under stereotype threat conditions [6].

### Stereotype threat in real world contexts

Our goal in this paper is to discuss one particularly important context in which stereotype threat is hypothesized to negatively impact women’s mathematics performance: authentic high-stakes tests (i.e., not in low-stakes laboratory tests, the context of the large majority of literature on stereotype threat). Stricker and Ward [7] investigated the extent to which stereotype threat plays a role in influencing women’s real-world mathematics achievement by manipulating the location of demographic questions on two authentic high-stakes tests: the 1996 Advanced Placement Calculus AB examination and the Computerized Placement Test, both qualifications intended for students seeking college credit or placement [8]. Half the participants were asked to state their ethnicity and gender before answering any questions, and half gave their ethnicity and gender after answering the questions. Their hypothesis was that asking participants to state their gender before tackling the questions would increase the saliency of gender, and therefore provoke stereotype threat among the women in the sample. In contrast, they reasoned, moving these demographic questions to the end of the examination would reduce the chance of stereotype threat impacting women’s performance.

Stricker and Ward [7] found the hypothesized significant manipulation-by-gender interaction on the AP Calculus AB exam (we focus on the ‘formula score’, which represents the number of correct answers, adjusted for guessing). The women who were asked about their gender in advance performed worse than those who were asked afterwards, with the men showing a small trend in the opposite direction. However, Stricker and Ward also noted that the size of this effect was small (less than 10% of the variance in scores could be accounted for by this interaction effect), and therefore concluded that stereotype threat was not practically significant. A similar result was found for the reading comprehension component of the second standardized test Stricker and Ward studied, but not for the predicted mathematical components.

Danaher and Crandall [9] reanalyzed Stricker and Ward’s [7] data, and argued that their criterion for ‘practical significance’ was too conservative. They pointed out that changing the location of demographic questions on examination scripts would be inexpensive and therefore that any evidence of a non-zero effect, regardless of its size, had policy implications. They calculated that changing the location of demographic questions would increase the number of U.S. women receiving AP Calculus AB credit by more than 4700 per year, and wrote that this “would be the single most cost-effective action our country could take to increase girls’ performance on AP Calculus exams” (p. 1652). In response, Stricker and Ward [10] rejected Danaher and Crandall’s argument, suggesting that they had selectively focused on women and mathematics (the original paper had also investigated ethnicity and verbal ability) and that, in view of traditional effect size guidelines, their decision to interpret a statistically significant but small effect as not practically significant was justified.

In our view, Danaher and Crandall’s [9] argument is persuasive. Effects in education research found where standardized tests are the dependent measures are typically small [10–12]. Because of this, using guidelines for effect size interpretation developed in the context of psychology may lead to effective interventions being dismissed [13]. A citation analysis suggests that Danaher and Crandall’s interpretation is the more widely accepted: Scopus reports that Danaher and Crandall’s [9] reanalysis has received more citations than Stricker and Ward’s [10] original report (as of 21st May 2021). Furthermore, in the education literature, Danaher and Crandall’s [9] interpretation is often cited without mention of Stricker and Ward [e.g., 14–16] and, when both authors are cited, the fact that Stricker and Ward disagreed with Danaher and Crandall is not always highlighted [e.g., 17].

Our goal in this paper is to report a conceptual replication of Stricker and Ward’s [10] investigation of stereotype threat in an authentic high-stakes setting, using the analysis approach favored by Danaher and Crandall [9]. Such a replication is timely, as since Stricker and Ward’s [10, 11] debate with Danaher and Crandall [9], several researchers have questioned the reliability of lab-based stereotype threat research. One reason is that attempts to replicate Spencer et al.’s [5] original lab study have not always been successful [e.g., 18]. Stoet and Geary [19] reviewed 23 replication attempts, finding that only 55% had results consistent with Spencer et al.’s, and that half of these only had so when the researchers controlled for participants’ pre-existing mathematics achievement (an analytic choice not made by Spencer at al.).

Flore and Wicherts [6] pointed out that the lab-based literature on stereotype threat and mathematics has an excess of significant findings (more significant results than one would expect given the average statistical power of published studies). They investigated two possible reasons. First, earlier researchers may have engaged in *p*-hacking, by using questionable research practices (such as selectively including covariates) to obtain significant effects [20]. Second, the literature may be subject to publication bias, a phenomenon where articles which report significant results are more likely to be accepted for publication than those which do not [18]. Flore and Wicherts’s [6] meta-analysis of 47 lab studies that investigated stereotype threat and mathematics achievement found that publication bias might have “seriously distorted” the meta-analytic effect size estimate they derived from the literature. However, they found that questionable research practices such as *p*-hacking were not, on their own, sufficient to have created the effect. They left open the possibility that a combination of publication bias and questionable research practices may be present in the literature.

In sum, there is now some doubt about the reliability of the lab-based literature on stereotype threat. While lab studies, on average, report small effects in the same direction as Spencer et al.’s [5] original experiment, it is unclear whether this effect is robust or an artefact of publication bias. If stereotype threat effects cannot be robustly found in well-controlled lab studies, it seems unlikely that they could be found in authentic contexts such as real-world high-stakes tests.

The particular history of Danaher and Crandall’s [9] analysis means that the two factors identified by Flore and Wicherts [6], publication bias and *p*-hacking, are unlikely to apply. Stricker and Ward’s [7] original article was published despite the authors claiming that they had found no effect. This means that Stricker and Ward did not have the usual motivation for *p*-hacking (finding significant effects where none exist), as although they reported a statistically significant *p* value, they interpreted it as indicating an effect so small as to be practically unimportant. Similarly, if publication bias had played a role then, given its negative conclusions, Stricker and Ward’s original paper would not have made it into print. For these reasons, it seems unlikely that Flore and Wicherts’s concerns about the general literature apply to this particular article.

In sum, we believe that Stricker and Ward’s [7] study merits replication. It is an influential study about a topic of societal importance which is often cited in policy debates and the education literature [e.g., 14–17, 21]; the wider literature has cast a degree of doubt about the reliability of the theoretical mechanism proposed to underly the effect; and the dispute between Stricker and Ward [7, 10] and Danaher and Crandall [9] about how to legitimately interpret the study’s findings, means that if the observed effect were a false positive, it is unlikely to be due to publication bias or questionable research practices.

### The context of the study

Our study took place in the context of the UKMT Junior Mathematical Challenge (JMC), a 60-minute, multiple-choice competition consisting of 25 mathematical problems. The UKMT describes the challenge as encouraging “mathematical reasoning, precision of thought, and fluency in using basic mathematical techniques to solve interesting problems.” It is aimed at students across the UK, particularly those aged 11–13. Exactly which young people participate is decided by their schools; some enter whole year groups and others enter only certain classes. The questions on the JMC are intended to be accessible to students studying the English National Curriculum (and the other national curricula of the UK). The top-scoring 40% of participants are awarded Bronze, Silver and Gold certificates in the ratio 3:2:1. The JMC is also the first round of a suite of problem solving competitions for students of this age. The roughly 1200 highest scorers are invited to participate in the Junior Mathematical Olympiad and the next highest 8000 or so are invited to participate in the Junior Kangaroo.

**Fig 1:**
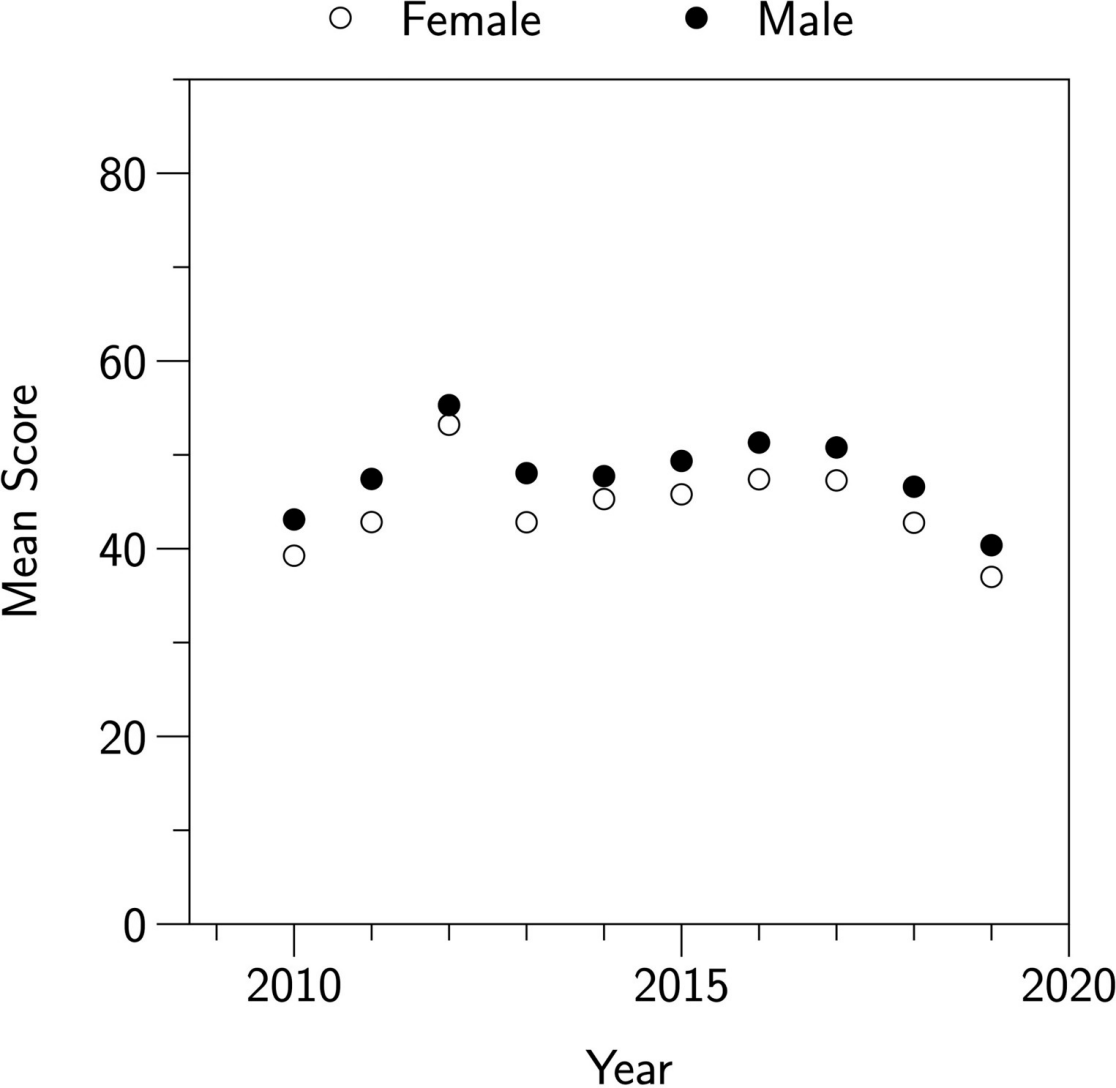
The mean scores of male and female participants in the JMC between 2010 and 2019. Because of the large samples (mean *N*/year = 247,566), error bars are not visible.

The JMC has a history of gender differences in outcomes, which have not been satisfactorily explained. Fig 1 shows the average scores for male and female participants since 2010. Across this period male participants scored between 0.10 and 0.24 standard deviations higher than female participants (mean 0.19). Because participants in the JMC are asked to state their demographic information, including gender, prior to answering questions we hypothesized that the stereotype threat effect discussed by Stricker and Ward [7, 10] and Danaher and Crandall [9] might be contributing to these disparities.

Clearly the JMC is a different context to the AP Calculus AB examination discussed by Stricker and Ward [7, 10] and Danaher and Crandall [9]. We return to this issue, and outline more precisely the differences between the respective contexts, in the discussion.

### Participants and procedure

Of the 2642 schools scheduled to take part in the 2019 JMC as of 14th December 2018, the largest 13 state schools (by expected number of entries) were approached by the UKMT to take part in the study and 6 agreed. We stopped inviting schools to participate after 6 agreed, as we felt this would provide adequate power to detect the hypothesized interaction (see sensitivity analysis below). The final sample consisted of students from 5 English state schools, because one school administered the test on the wrong day (and so their students were not eligible for JMC certificates, as they may have had prior access to the questions). Four of the five remaining schools were coeducational, one only taught girls. Three of the schools were selective, two were not. Students at the schools were entered in the JMC in the normal way, and took part on the same day as participants at other schools. Three schools (two selective) entered all their students in Years 7 and 8, two entered a selection of higher-attaining students to participate, based on their own assessment data.

The JMC is administered and invigilated by teachers (or other members of staff) in their own schools, typically in a school hall. The UKMT sends detailed instructions to each participating school and requires that these are followed. Our instructions asked invigilators to explain to students that they had the opportunity to contribute to a research study designed to help the UKMT improve its competitions in the future, and that their answer sheet may be different to those around them. No information was given to participants about the purpose of the study in advance.

Two different versions of the (optical mark recognition) answer sheet were distributed to schools. The versions were supplied to schools in a random order, and teachers were asked to ensure that they were handed out in order to students according to where they were sitting (which followed the school’s normal policy for examinations). This ensured that our two experimental groups were formed randomly.

Each answer sheet had four sections. Section 1 of the gender-first version asked participants to state their first name, surname and gender; Section 2 asked them to give their answers to the JMC questions (all multiple choice questions supplied on a separate sheet); Section 3 asked them to state their school’s name and their year group; Section 4 asked participants to state whether or not they gave consent for their data to be included in the analysis. The gender-last version of the answer sheet switched the order of Sections 1 and 3. Sections 1 and 3 were always on different sides of the answer sheet, and participants were instructed not to turn over their answer sheets. We used the UKMT’s standard phrasing for all questions, which in the case of gender was “Please indicate your gender by putting a solid line through one of the options”, with the options being “female”, “male” and “unspecified” in that order. (The wording of the questions used to gather demographic information on the JMC has changed over the years. In the early years of the competition participants were asked for their “sex” with three options: female, male, unspecified. In the past 20 years the wording was changed to ask participants for their “gender” but with the same three options. Since we conducted the study the UKMT have changed the text of the question used to ask for students’ gender. It now reads: “GENDER (optional): [] Boy or young man, [] Girl or young woman”, and a third option with a blank box to allow participants to self-describe their gender. For clarity, in the remainder of the paper, we use the terms “male” and “female” to refer to participants who identified themselves as such on their answer sheet.) Participants were given five minutes to complete Section 1, one hour for Section 2, and five minutes for Sections 3 and 4.

As noted above, in Section 4 test takers were asked whether or not they gave consent for their data to be used in the study. All those who explicitly refused consent (*N* = 265), or who failed to answer this question (*N* = 94) were omitted from the analysis, but were nevertheless eligible for achievement certificates from the UKMT. Importantly, there was no significant association between whether or not participants consented for their data to be analyzed, and which answer sheet they had received (gender-first or gender-last), χ^2^(1) = 1.351, *p* = .245, suggesting that different consent levels between conditions was not a threat to the validity of our results.

As preregistered, after each school had returned their students’ answer sheets, they were asked to confirm that they followed our instructions to the letter. Two schools provided a list of 44 scripts from students who did not follow the instructions (e.g., they turned over their answer sheet during the Section 1 phase). Although our preregistration had only anticipated excluding entire schools where the invigilation had not proceeded in line with the instructions, we felt it appropriate to exclude these 44 participants. We did not preregister any other exclusions, but it was necessary to exclude 23 participants who did not report their gender (20 did not answer the question, 3 chose “unspecified”). This left 1169 participants: 719 females and 450 males. A sensitivity analysis indicated that this sample size gave us 80% power to detect a gender by answer-sheet version (gender-first/gender-last) of *η*^2^ = 0.0065 [22].

The study was approved by the Loughborough University Ethics (Human Participants) Subcommittee (reference R18-P140). Our analysis plan was preregistered at AsPredicted.org (#21450) prior to data collection, and can be inspected at https://aspredicted.org/hf3jc.pdf. The 2019 JMC examination paper, the two experimental response forms, the response form used by challenge participants not involved in the study, the invigilator’s instructions, and the raw data and analyses scripts are available at https://doi.org/10.17605/OSF.IO/UMJ4H.

## Results: Preregistered analyses

To create our dependent variable we used the standard, and longstanding, JMC scoring system, which awarded 5 points for each correct answer to the first 15 questions, 6 points for each correct answer to the last 10 questions. One point and 2 points were deducted for incorrect answers to questions 15–20 and questions 21–25 respectively subject to a minimum total score of zero. Participants’ scores varied from 0 to 110, and their mean, *M* = 44.9, SD = 19.7, was slightly higher than the overall average, *N* = 251,064, *M* = 38.71, SD = 19.2, *t*(1168) = 10.7, *p* < .001, *d* = 0.312.

**Fig 2:**
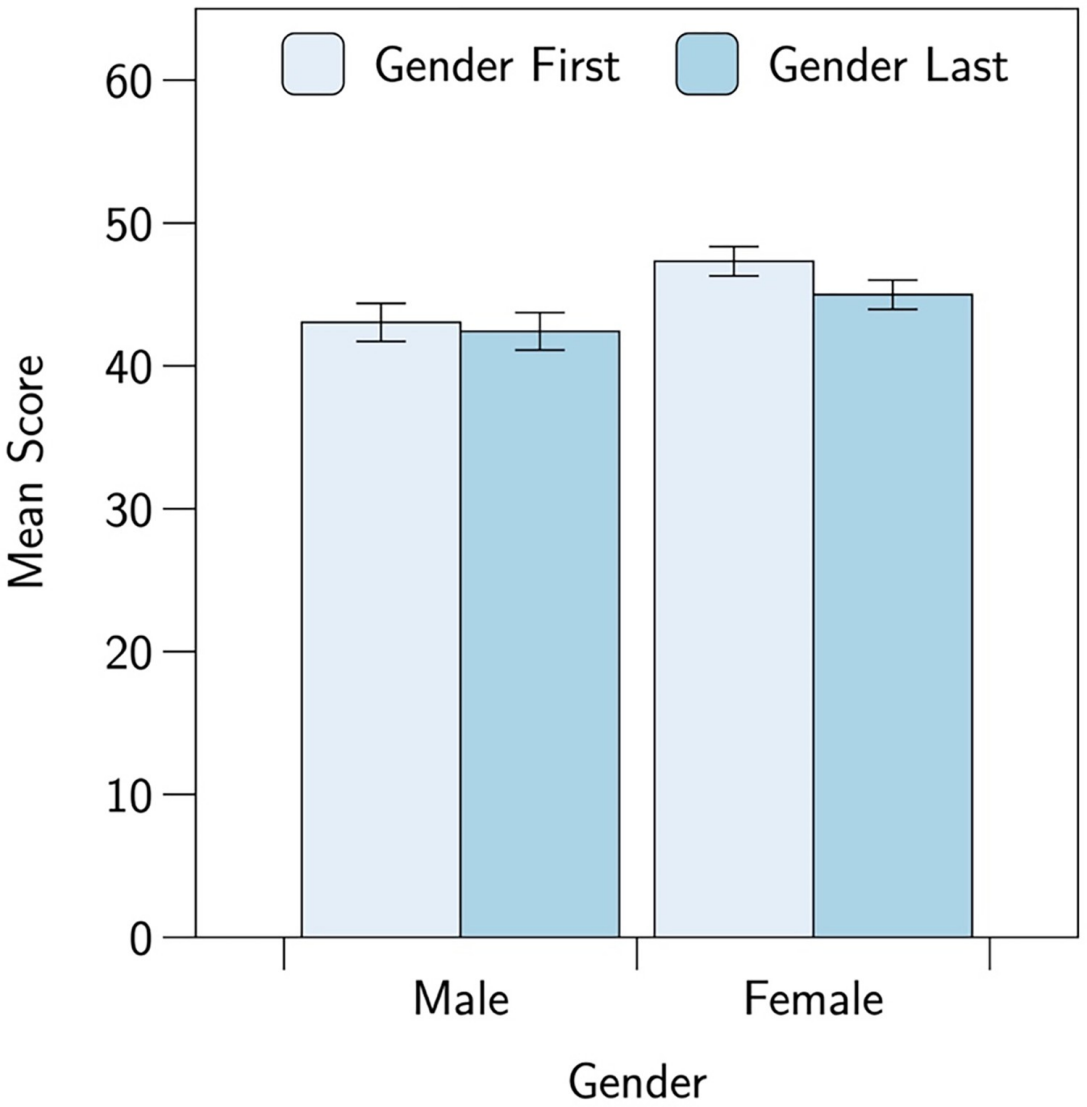
A plot showing participants’ mean scores, split by gender and answer sheet version. Error bars show ±1 SE of the mean.

Participants’ mean scores, split by answer-sheet version and gender, are shown in Fig 2. As stated in our preregistration, these scores were subjected to a 2 (version) by 2 (gender) between-subjects Analysis of Variance (ANOVA). This revealed a significant main effect of gender, *F*(1,1165) = 8.410, *p* = .004, *η*^2^ = .007, which reflected that female participants had a higher mean score than male participants, 46.2 versus 42.7, *d* = 0.177. There was no significant main effect of version, *F*(1,1165) = 1.586, *p* = .208, *η*^2^ = .001, (means 45.7, 44.0, *d* = 0.091. Crucially, we did not find the hypothesized version-by-gender interaction effect, *F*(1, 1165) = 0.525, *p* = .469, *η*^2^ = .000. Indeed, contrary to the prediction of the stereotype threat account, female participants in the gender-first condition had slightly (but non-significantly) higher scores than those in the gender-last condition, 47.3 v 45.0, *t*(717) = 1.61, *p* = .108, *d* = 0.120.

To be consistent with Stricker and Ward [7], our primary preregistered analysis involved performing an ANOVA. However, we also ran a multilevel analysis to take account of between-school variation. We compared models with (i) random intercepts (where intercepts were able to vary across schools) and (ii) random intercepts and slopes (where both intercepts and slopes were able to vary across schools). Allowing intercepts to vary across schools yielded a significantly better fit (BIC = 10037.65) than a model where intercepts were identical across schools (BIC = 10298.66), χ^2^(2) = 268.1, *p* < .001. However, allowing slopes to vary across schools did not significantly improve the fit of the model that included version, gender and the version-by-gender interaction (both BICS = 10047.86), χ^2^(2) = 0.00, *p* = 1. In this model (in which gender was coded 0 for males, 1 for females; and version was coded 0 for gender-first and 1 for gender-last), neither version, *b* = -0.902, *t*(1161) = -0.551, *p* = .582, nor gender, *b* = -2.99, *t*(1161) = -1.863, *p* = .063, nor the version-by-gender interaction effect, *b* = -1.04, *t*(1161) = -0.499, *p* = .618, were significant predictors of participants’ scores (intercept *b* = 46.95, *t*(1161) = 8.96, *p* < .001). In sum, analyzing the data in this fashion again provided no evidence of the hypothesized version-by-gender interaction.

Finally, we conducted a preregistered Bayesian version of our main ANOVA [23]. This required us to specify a model for the null hypothesis. As specified in our preregistration, we ran two analyses, with Cauchy prior widths of 0.2 and 0.5. Both analyses provided strong support for the model that only included gender as a predictor over the model which captured the predicted stereotype threat effect (i.e. the model which included gender, version and the version-by-gender interaction effect), BF_01_s = 8.123, 46.052 respectively.

### Results: Exploratory analyses

To explore whether our inclusion of a single-gender school in the sample effected the results (perhaps, for example, students at single-gender schools are not as affected by societal stereotypes as those at coeducational schools [cf. 24]), we conducted an exploratory analysis with the 329 participants from this school omitted. This resulted in an essentially identical pattern of results. In particular we again found no significant version-by-gender interaction effect, *F*(1,836) = 0.059, *p* = .809, *η*^2^ = .000.

To explore whether or not our decision to use the standard method of scoring the JMC affected the results, we conducted the primary ANOVA analysis again using (i) number of problems answered correctly and (ii) percentage accuracy (number of problems answered correctly as a percentage of problems attempted) as dependent variables. Our primary conclusions remained for both these dependent variables. Specifically, neither version-by-gender interaction effects with these two dependent variables was significant: number correct, *F*(1,1165) = 0.253, *p* = .615, *η*_*p*_^2^ = .000; percentage accuracy, *F*(1,1165) = 0.326, *p* = .568, *η*_*p*_^2^ = .000.

In sum, we found no evidence in favor of the hypothesis that female participants scored lower when they received the gender-first version of the answer sheet in any of our analyses, and a Bayesian analysis provided strong evidence against this hypothesis.

## Discussion

Danaher and Crandall’s [9] reanalysis of Stricker and Ward’s [7] study of stereotype threat in real-world conditions suggested that moving the location of demographic questions in test answer sheets could contribute to reducing the impact of stereotype threat on women’s performance. We conducted a conceptual replication of Stricker and Ward’s study in the context of the UKMT JMC and found no evidence for this effect.

Are there any reasons to doubt the validity of our findings? There are at least two ways in which our sample could be said to be unrepresentative. First, the students in our sample had significantly higher scores than the overall average in the 2019 JMC. Second, although there was a significant difference in overall male and female performance in the 2019 JMC (shown in Fig 1), in our sample we found the reverse pattern: our female participants significantly outperformed our male participants. We discuss each of these factors in turn.

Could the relatively high performance, in comparison with the wider population, of students in our sample account for us not having found the hypothesized stereotype threat effect? We think not. In fact, some earlier findings suggest that this factor should *increase* the size of the effect. For example, Pronin, Steele and Ross [25] found that female students with higher calculus GPAs and who identified more strongly with mathematics, were more impacted by a stereotype threat manipulation than those with lower calculus GPAs, or lower levels of mathematics identification. In other words, because our sample consisted of relatively successful mathematics students, we might expect the stereotype threat to be magnified.

Might the small female advantage found in our sample, compared to the small male advantage found nationally, account for the lack of a stereotype effect in our data? Again, we doubt this. This difference was driven by the inclusion of a high achieving girls-only school in our sample. This school had the highest mean score of any which participated. When this school was excluded from our analysis, we found a small male advantage consistent with the overall picture, *t*(838) = 2.391, *p* = .017, *d* = 0.165. As noted above, our substantive conclusions remain if the analysis is conducted on this restricted sample (*N* = 840).

So what accounts for the difference between our results and those reported by Danaher and Crandall [9]? Does this failure to replicate indicate that their finding was a false positive? Not necessarily. Certain factors are theorized to increase the effect of stereotype threat [26]. Specifically, stereotype threat is thought to be more prominent (i) when the test is more difficult [27, 28]; (ii) when stereotyped groups show higher levels of domain identification with the content of the test [28]; (iii) when (in the context of mathematics/gender stereotype threat) participants have higher levels of mathematics anxiety [17, 29]; and (iv) when participants consider membership of the stereotyped groups to be an important part of their identity [30]. What can be said about these factors in the context of our study?

### Test difficulty

As noted above, our sample scored slightly above the overall average for the 2019 JMC. However, we should not conclude from this that the test was not challenging for our sample. The mean number of correct answers offered by participants was 9.5 (from a total of 25 questions), and the mean accuracy (expressed as a percentage of attempted questions) was 49%. These figures suggest that it is likely that our sample found the 2019 JMC paper to be challenging.

### Domain identification

We do not have direct evidence about the extent to which our participants identified with mathematics as a domain, or the extent to which they were anxious about mathematics. However, the structure of our dataset does allow us to approach these questions, albeit indirectly. Specifically, as noted in the introduction, some schools enter their entire cohort into the JMC while others restrict entry to pupils in classes for higher attaining students (the large majority of English schools teach mathematics in attainment groups). In our sample, two of the five participating schools restricted entry in this fashion. On the assumption that female participants from high-prior-attainment classes were more likely to have higher domain identification than female participants from low-prior-attainment classes, we repeated our primary analysis, restricted to participants from just these two schools (*N* = 660). There was again no significant gender by version interaction, *F*(1, 656) = 0.00, *p* = .996, *η*^2^ = .000. Critically, the female participants from these schools had very similar mean scores for the two version conditions: gender-first mean 44.4, gender-last mean 43.1, *t*(313) = 0.661, *p* = .509, *d* = 0.07. In sum, when our analysis was restricted to a subsample that we might expect to show higher levels of domain identification we again found no evidence of the hypothesized stereotype threat effect. Of course, prior attainment is not a perfect proxy for domain identification, so this analysis should not be considered definitive.

### Mathematics anxiety and gender identification

Unfortunately we do not have any evidence concerning the extent to which the female participants in our study were anxious about mathematics, or whether they considered gender to be an important part of their self-identity, so cannot speak to these moderators.

There are also differences between the context of our study and the context of Stricker and Ward’s [7] that must be highlighted. Indeed, Schoenfeld [31] pointed out that direct and conceptual replications can serve different purposes. He argued that while direct replications help us guard against ‘statistical accidents’ (Type I errors where significant results are obtained in the sample despite there being no true effect in the population), a main goal of conceptual replications is to help guard against results that do not generalize beyond their initial contexts. Our study was a conceptual replication, not direct replication, and our context differs from the original in significant ways. We note five: (i) JMC participants are younger than AP Calculus AB participants (early high school rather than late); (ii) the JMC takes place in the UK rather than the US; (iii) our study took place in 2019 rather than 1996; (iv) the JMC is likely a lower stakes examination than the AP Calculus AB examination; and (v) the ethnic makeup of our sample may have been different to Stricker and Ward’s.

Might one of these factors have caused our failure to replicate? We doubt either of the first two are responsible. First, the stereotype threat effect has been observed in young children as well as college students [e.g., 32], so there is no reason to suppose that early high school students would not be affected. Second, analyses of cultural differences in implicit associations between gender and science have found that the UK and US have similar profiles [33], suggesting that both countries have similar societal stereotypes with respect to gender and mathematics.

Drawing on evidence from draw-a-scientist studies, Miller, Nolla, Eagly and Uttal [34] reported evidence that the children’s gender-science stereotypes may have reduced over time, at least in the US. This result perhaps lends credence to the possibility that the fact that our study took place two decades after Stricker and Ward’s [7] might account for the different results. Perhaps gender-mathematics stereotypes are simply not as strong as they were in the 1990s. If this account were correct we would indeed expect to see reduced stereotype threat effects.

The fourth difference noted above concerns the extent to which the JMC can be considered a high-stakes setting. There are several reasons to suppose that participants do regard the JMC as being an important test. First, the test takes place in formal examination conditions (i.e. in an examination hall outside of normal classes). Second, the JMC is a national competition that is used to award certificates and select participants for subsequent competitions. Third, schools often enthusiastically highlight strong performances on the JMC by their students to parents, and experience suggests that students commonly discuss their participation in the JMC on the ‘personal statement’ section of their applications to study at university. Nevertheless, it seems plausible to suppose that the stakes involved in the JMC are not as high as the AP Calculus AB examination, which is used by some students to gain college credit.

Finally, it is worth noting that–in line with the UKMT’s normal practice–we did not enquire about the ethnicity of the participants in our sample. In Stricker and Ward’s [7] study there was a significant version-by-gender-by-ethnicity interaction (for the formula score dependent variable on the AP Calculus AB examination), with some suggestion that the reduction in performance in the gender-first condition compared to the gender-last condition was greater for black female participants than white female participants. In the absence of ethnicity data for our sample we are unable to explore this potential factor further.

Despite these considerations, the other possibility highlighted by Schoenfeld’s [31] discussion–that the original result could be a ‘statistical accident’–should not be dismissed. The disagreement between Stricker and Ward [7, 10] and Danaher and Crandall [9] about the correct interpretation of the original findings, coupled with subsequent growing doubts about how robust the stereotype threat effect is more generally, suggests that we should take seriously the possibility that Danaher and Crandall’s conclusions were based on a false positive.

While we cannot definitively determine whether our results differ from Danaher and Crandall’s [9] because of a ‘statistical accident’, or because our context was different, we can conclude, in line with Stricker and Ward [10], that the ‘silver bullet’ intervention of relocating demographic questions on test answer sheets is unlikely to be effective in all contexts. Instead, we suggest that much more deep-seated interventions are required if we are to successfully address systemic gender inequities in mathematics.
